# Supplementary material for: Machine-learning-assisted analysis of transition metal dichalcogenide thin-film growth
Source: Nano Converg. 2023 Feb 20;10:10. doi: 10.1186/s40580-023-00359-5 (PMC9941396; doi:10.1186/s40580-023-00359-5)
Supplement: Supplementary file 1 — Additional file 1: Figure S1. XPS spectra of 3UC ReSe2 thin film. (a) Wide scan, and narrow scans in (b) Re 4f, and (c) Se 3d peak. Atomic ratio of Se/Re is about 2.01. Figure S2. Fraction variance of six principal components of PCA in 3UC ReSe2. Figure S3. PCA result in ReSe2 thin films with different thicknesses. (a,c,e) Four principal components of the RHEED video and (b,d,f) their corresponding score plots. Figure S4. Reconstruction of RHEED video for identifying the film thickness. Intensity plot of the original RHEED video and the modified RHEED video in ReSe2 thin films with different thicknesses; (a) 2UC, (b) 4UC, and (c) 5UC. Blue and orange lines denote the (0,0) and (2,0) diffraction streaks of the ReSe2 thin film (shown in the inset of Fig. 4(a)), respectively. [file 40580_2023_359_MOESM1_ESM.docx]

**Supplementary information**

**Machine-learning-assisted growth analysis of transition metal dichalcogenide thin-film growth**

Hyuk Jin Kim^1, #^, Minsu Chong^1, #^, Tae Gyu Rhee^1,2^, Yeong Gwang Khim^1,2^, Min-Hyoung Jung^3^, Young-Min Kim^3^_,_ Hu Young Jeong^4^, Byoung Ki Choi^1,5^, Young Jun Chang^1,2,*^

*^1^ Department of Physics, University of Seoul, Seoul, 02504, Republic of Korea,*

*^2^ Department of Smart Cities, University of Seoul, Seoul, 02504, Republic of Korea,*

*^3^ Department of Energy Science, Sungkyunkwan University (SKKU), Suwon, 16419, Republic of Korea,*

*^4^ Graduate School of Semiconductor Materials and Devices Engineering, Ulsan National Institute of Science and Technology (UNIST), Ulsan, 44919, Republic of Korea,*

*^5^ Advanced Light Source (ALS), E. O. Lawrence Berkeley National Laboratory, Berkeley, California 94720, USA*


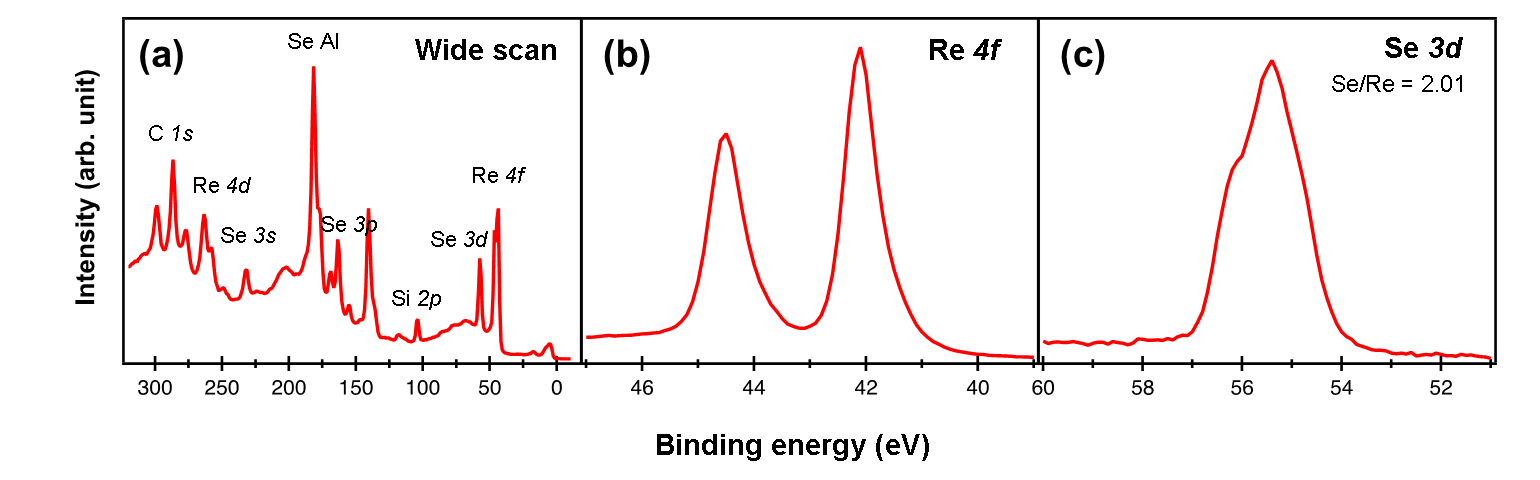


**Figure S1.** XPS spectra of 3UC ReSe_2_ thin film. (a) Wide scan, and narrow scans in (b) Re *4f*, and (c) Se *3d* peak. Atomic ratio of Se/Re is about 2.01


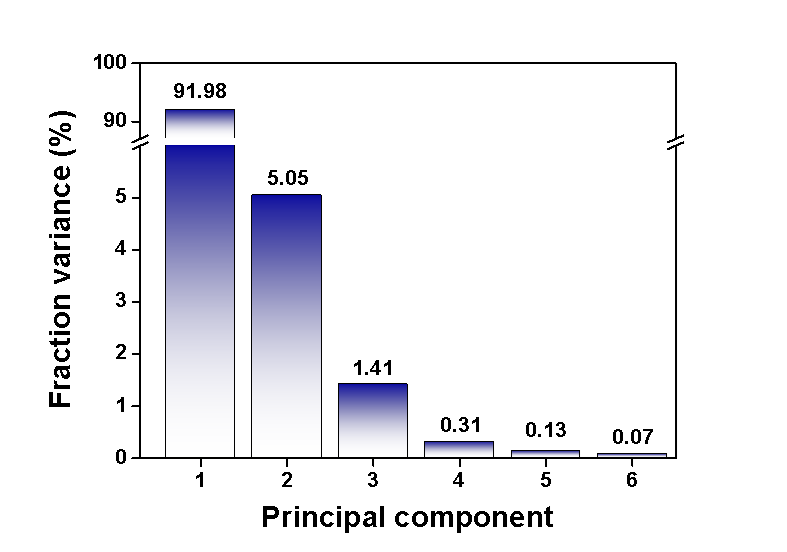


**Figure S2.** Fraction variance of six principal components of PCA in 3UC ReSe_2_.


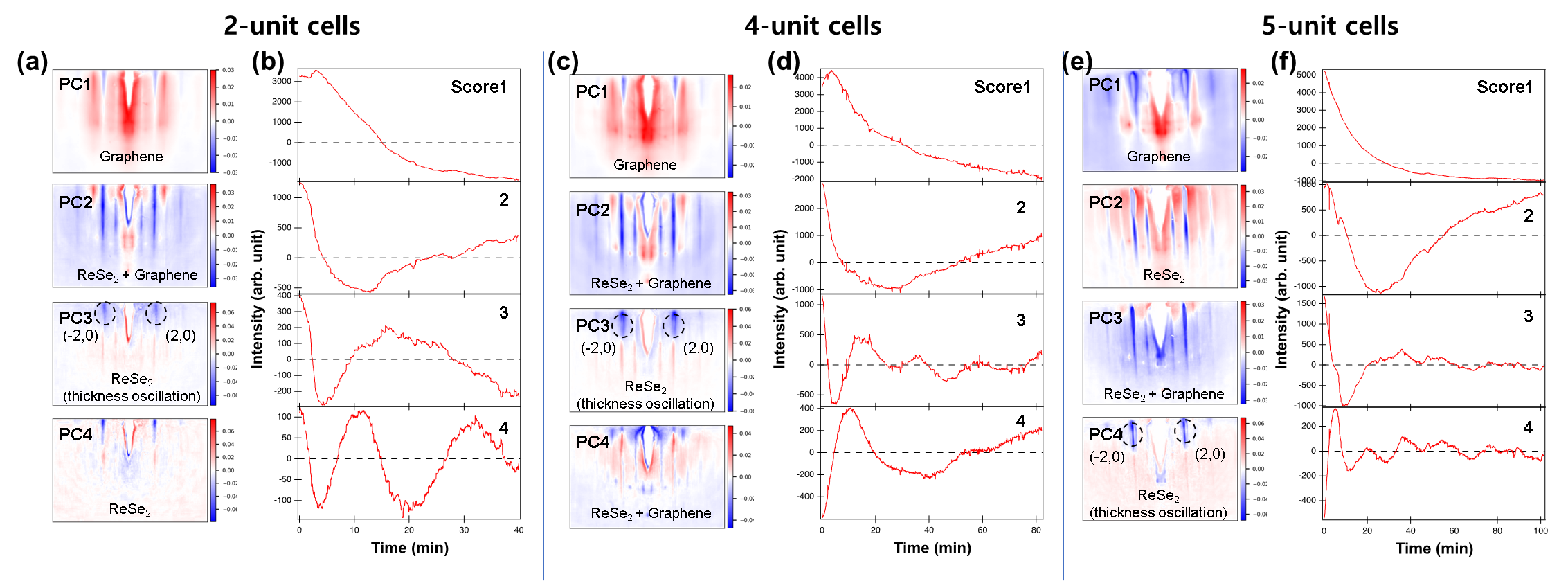


**Figure S3.** PCA result in ReSe_2_ thin films with different thicknesses. (a,c,e) Four principal components of the RHEED video and (b,d,f) their corresponding score plots.


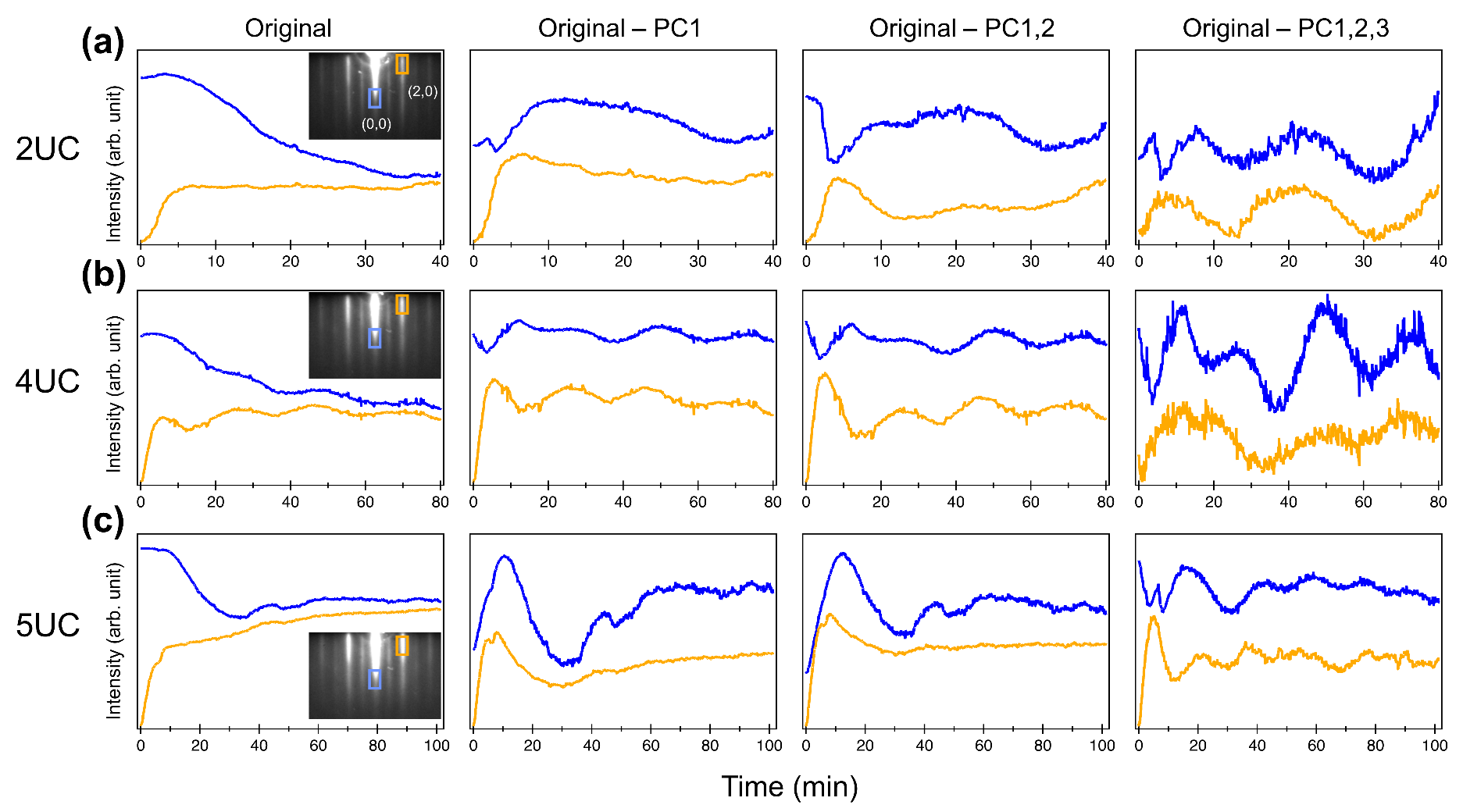


**Figure S4.** Reconstruction of RHEED video for identifying the film thickness. Intensity plot of the original RHEED video and the modified RHEED video in ReSe_2_ thin films with different thicknesses; (a) 2UC, (b) 4UC, and (c) 5UC. Blue and orange lines denote the (0,0) and (2,0) diffraction streaks of the ReSe_2_ thin film (shown in the inset of Fig. 4(a)), respectively.
